# Statement of Retraction: Electrochemical and hot corrosion behaviour of steel reinforced with AlSiBeTiV high entropy alloy using friction stir processing

**DOI:** 10.1080/14686996.2025.2552539

**Published:** 2025-09-04

**We, the journal office and Publisher of *Science and Technology of Advanced Materials (STAM)*, have retracted the following article:**

Sundaram, R., Nachimuthu, R., Sivanandam, A. K., & Natarajan, J. (2024). Electrochemical and hot corrosion behaviour of steel reinforced with AlSiBeTiV high entropy alloy using friction stir processing. Science and Technology of Advanced Materials, 25(1). https://doi.org/10.1080/14686996.2024.2320083

Following publication, the publisher was contacted by a third party with concerns about the integrity of the data, specifically:

  1. Apparent similarities between Figure 3a in the above article and the image in Figure 2d of:

Singh, A., Akhil, U.V., Kishan, S.N., Anoosa Sree, R., Radhika, N., & Rajeshkumar, L (2024). Synthesis of a novel AlBeSiTiV light weight HEA coating on SS316 using atmospheric plasma spray process. Heliyon, 10(16). https://doi.org/10.1016/j.heliyon.2024.e35999

  2. The XRD spectra plotted in Figure 15 appear to have the same background noise.

When contacted by the journal for an explanation, the authors were unable to address the concerns raised. As a result of these concerns, the STAM office no longer has confidence in the validity of the findings in the article, and therefore we are retracting the article from the journal. The corresponding author listed in this publication has been informed. The author does not agree with the retraction.

We have been informed in our decision-making by our editorial policies and the COPE guidelines.

The retracted article will remain online to maintain the scholarly record, but it will be digitally watermarked on each page as ‘Retracted’.

